# Reinforcement Learning for Central Pattern Generation in Dynamical Recurrent Neural Networks

**DOI:** 10.3389/fncom.2022.818985

**Published:** 2022-04-08

**Authors:** Jason A. Yoder, Cooper B. Anderson, Cehong Wang, Eduardo J. Izquierdo

**Affiliations:** ^1^Computer Science and Software Engineering Department, Rose-Hulman Institute of Technology, Terre Haute, IN, United States; ^2^Computational Neuroethology Lab, Cognitive Science Program, Indiana University, Bloomington, IN, United States

**Keywords:** CTRNN, dynamical neural networks, reinforcement learning, neuromodulatory reward, dynamic synapse, lifetime learning

## Abstract

Lifetime learning, or the change (or acquisition) of behaviors during a lifetime, based on experience, is a hallmark of living organisms. Multiple mechanisms may be involved, but biological neural circuits have repeatedly demonstrated a vital role in the learning process. These neural circuits are recurrent, dynamic, and non-linear and models of neural circuits employed in neuroscience and neuroethology tend to involve, accordingly, continuous-time, non-linear, and recurrently interconnected components. Currently, the main approach for finding configurations of dynamical recurrent neural networks that demonstrate behaviors of interest is using stochastic search techniques, such as evolutionary algorithms. In an evolutionary algorithm, these dynamic recurrent neural networks are evolved to perform the behavior over multiple generations, through selection, inheritance, and mutation, across a population of solutions. Although, these systems can be evolved to exhibit lifetime learning behavior, there are no explicit rules built into these dynamic recurrent neural networks that facilitate learning during their lifetime (e.g., reward signals). In this work, we examine a biologically plausible lifetime learning mechanism for dynamical recurrent neural networks. We focus on a recently proposed reinforcement learning mechanism inspired by neuromodulatory reward signals and ongoing fluctuations in synaptic strengths. Specifically, we extend one of the best-studied and most-commonly used dynamic recurrent neural networks to incorporate the reinforcement learning mechanism. First, we demonstrate that this extended dynamical system (model and learning mechanism) can autonomously learn to perform a central pattern generation task. Second, we compare the robustness and efficiency of the reinforcement learning rules in relation to two baseline models, a random walk and a hill-climbing walk through parameter space. Third, we systematically study the effect of the different meta-parameters of the learning mechanism on the behavioral learning performance. Finally, we report on preliminary results exploring the generality and scalability of this learning mechanism for dynamical neural networks as well as directions for future work.

## 1. Introduction

Adaptive behaviors, or beneficial changes to behavior over time, are a hallmark of living organisms. Partly, these behaviors are changed or acquired over evolutionary time, through the evolution of the body and the basic structure of the nervous system, but also partly based upon experiences during individual lifetimes. Under simple enough circumstances, behaviors may appear instinctive—an organism responds in a predictable way to a given stimulus as a result of its evolved characteristics. However, most, if not all, living organisms can change their behavior during their lifetime based on experience (Sasakura and Mori, 2013; Dussutour, 2021; Shapiro, 2021). As a result, many living organisms can adaptively improve their performance on some behavioral tasks during their lifetime. Despite several decades of study, how learning arises within an organism's lifetime is still poorly understood (Sweatt, 2016; Schaefer et al., 2017). Although learning during a lifetime is widely considered a job performed by the synapse, its exact contribution remains obscure (Mozzachiodi and Byrne, 2010; Humeau and Choquet, 2019). Despite the undisputed importance of synaptic plasticity for brain function, computational models of embodied and situated neural systems have not yet produced autonomous lifetime learning by merely including synaptic plasticity.

The theoretical study of learning in neural models outside of computational neuroscience has had a great impact within its exploration of the space of possibilities (Abbott, 2008; van Ooyen, 2011; O'Leary et al., 2015). There has been impressive progress on training increasingly large artificial neural networks to solve certain tasks by relying on the use of gradient-descent methods, like backpropagation (Bengio et al., 2015). These methods require time-invariant and differentiable neural networks, which has allowed the machine learning community to thrive while focusing primarily on feedforward discrete neural networks (Glaser et al., 2019). Biological neural circuits are, however, highly recurrent, non-linear, and dynamic, and hence undifferentiable (White et al., 1986; Franconville et al., 2018; Litwin-Kumar and Turaga, 2019). While dynamic recurrent neural networks have emerged as powerful and versatile tools of neuroscience research (Harvey et al., 2005; Barak, 2017), how they can be made to acquire behaviors through rewards is still poorly understood (Beer and Barak, 2019).

In the field of computational neuroscience, most work focuses on the neural activity patterns produced by either biophysical models of a single neuron, small neural circuits, isolated regions of a nervous system, or large-scale models that reproduce certain statistical properties of their biological counterparts (Gerstner et al., 2012; Kim et al., 2014; Brunel, 2016; Williamson et al., 2019; Vyas et al., 2020), with and without synaptic plasticity. By focusing on neural activity in a vacuum, without taking into consideration the interactions of the nervous system with the organisms' body and its environment (Chiel and Beer, 1997; Tytell et al., 2011; Krakauer et al., 2017), the study of the relationship between synaptic plasticity and lifetime learning has been limited in scope or altogether absent.

In the fields of computational neuroethology, evolutionary robotics, and artificial life, there have been efforts to embed and situate nervous systems within bodies and environments. The nervous systems are modeled as dynamic recurrent neural networks, and the goal is study how these systems can generate adaptive behaviors of interest (Nolfi and Floreano, 2000; Harvey et al., 2005; Beer and Chiel, 2008; Bongard, 2013; Doncieux et al., 2015). However, because the goal of much of this work is to better understand how behavior is generated by these complex brain-body-environment systems, it has focused on the use of evolutionary algorithms and other similar stochastic search algorithms to find the parameters of the dynamical recurrent neural networks that produce the behavior of interest (Beer and Gallagher, 1992; Lehman and Miikkulainen, 2013; Stanley et al., 2019). Therefore, the learning in these systems occurs over evolutionary timescales; through a process of selection, inheritance, and mutation; not through dynamic changes in the parameters of the artificial organism as a result of experience during their lifetime. There has been a relatively large body of work within this field that has provided mechanisms of plasticity to the neural circuits and used evolution to fine-tune their properties (for a review, see Soltoggio et al., 2017). However, most of this work has focused on demonstrating that the evolutionary algorithm can find plasticity mechanisms that allow the neural circuit to perform a behavior of interest. Although it is important to understand that neural circuits can cope with plastic synapses and still produce behaviors robustly without disruptions; the focus has not been on demonstrating that these plasticity rules allow the dynamical recurrent neural circuits to learn new tasks based on experience. Importantly, by evolving neural circuits to produce not a particular behavior but to perform well on a learning task, there have been several demonstrations that learning behavior can be produced with and without synaptic plasticity (Yamauchi and Beer, 1994; Phattanasri et al., 2007; Izquierdo et al., 2008). Despite great progress in linking behavior to neural and synaptic dynamics, we do not yet have a good understanding of how dynamic recurrent neural networks can be coupled with synaptic plasticity mechanisms to produce learning behavior.

Recently, Wei and Webb (2018a,b) proposed a biologically-plausible reinforcement learning rule that can be applied to non-differentiable neural networks. Most non-differentiable neural network models assume static activation functions and synaptic weights during their simulation, with an optimization process, frequently an evolutionary algorithm, making changes between simulations. In contrast, the learning rules in their work are inspired by the dynamics in neurons' post-synaptic regions, which include the trafficking of neurotransmitter receptors. This trafficking results in fluctuations in the effective synaptic strengths of the network. Modeling this allows the exploration of adjacent values for synaptic weights, just as they are explored in biological neural circuits. A global reward signal is used as a control mechanism, which allows a network's synaptic weights to change with reward through an explore/exploit strategy, wherein the exploration becomes wider with negative rewards and narrower with positive rewards. The idea of a global reward (or reward prediction error) signal is common within reinforcement learning and has been hypothesized to be encoded by neuromodulatory signals such as dopamine (Glimcher, 2011; Dayan, 2012).

Our goal here is to extend the original work on these biologically-plausible reinforcement learning rules by applying them to a family of dynamical recurrent neural networks called continuous-time recurrent neural networks (CTRNNs) that have been studied in great detail (Beer, 1995, 2006) and that have been employed extensively in the evolutionary robotics, adaptive behavior, and computational neuroethology literature (Beer, 1997; Blynel and Floreano, 2002; Izquierdo and Lockery, 2010). We first develop a simple pattern generation task and examine in some detail the learning dynamics in a single successful simulation of lifetime learning. Next, we repeat the learning experiment multiple times in order to compare the performance of the reinforcement learning rule to two standard ways to search a space: hill-climbing and random search, with the expectation that the lifetime learning mechanism will perform better than random search and potentially as good as hill-climbing. Then, we examine the performance of the learning method systematically in relation to the initial distance the solution is away from a successful solution. The success of the reinforcement learning rules are dependent upon multiple metaparameters that play important roles. To gain insight into these roles, we systematically vary a few key metaparameters of the reinforcement learning rules to report on their relative efficacies. Finally, we also expand the fluctuation mechanism beyond the synaptic strengths to include fluctuations of the biases of the neurons to demonstrate that these same reinforcement learning rules can be successfully applied more ubiquitously to additional parameters of the dynamical system. This also allows us to test the ability to train networks of different sizes from random starting configurations, enabling us to present preliminary results regarding the scalability of the learning rules.

In what follows, we will first explain the neural model, training strategies, and task used to conduct experiments. Next, we will report on results evaluating the reinforcement learning rules and comparing them to our baseline models. This will then lead into reporting on a systematic study of metaparameters as well as our findings in relation to scalability and generalization. Finally, we discuss the major findings and implications of these results and then suggest several fruitful directions for future research in this area.

## 2. Methods

This section introduces the neural model, reinforcement learning rules applied to the neural model, the task, baseline learning strategies, performance measures, and experimental setup. We start by introducing the dynamical CTRNN model. Next, we explain how we adapted the previously published model to create the Reinforcement Learning CTRNN (RL-CTRNN). We explain the rhythmic pattern generation task used to measure learning ability and then describe the baseline learning strategies used for comparison with the RL-CTRNN. After exploring the learning strategies, we provide further details on how we calculate the reward signals for the RL-CTRNN. Finally, we explain the approach to conducting experiments systematically.

### 2.1. Dynamic Recurrent Neural Network Model

In this work, we extend the application of the reward-modulated plasticity rules to Continuous-Time Recurrent Neural Networks (CTRNNs) (Beer, 1995), where each neuron's state is governed by the canonical state equation:


(1) $$\begin{array}{r} \tau_{\text{i}}{ẏ}_{i}=-y_{i}+\overset{N}{\underset{j=0}{\sum }}w_{ji}\;\sigma \left(y_{j}+\;\theta_{j}\right)+I \end{array}$$

where *y*_*i*_ is the state of each neuron, τ_*i*_ is the time constant, *w*_*ji*_ is the weight of the connection between the *j*_*th*_ and *i*_*th*_ neuron, θ_*j*_ is a bias term, *I* is the injected current (which, for this work, is fixed at zero and removed from subsequent equations) and σ is the standard logistic activation function:


(2) $$\begin{array}{r} \sigma \left(x\right)=\;\frac{1}{1\;+\;e^{-x}} \end{array}$$

While CTRNNs can, in principle, approximate the trajectories of any smooth dynamical system (Funahashi and Nakamura, 1993), in this work, we start by considering the weights of edges as analogous to synaptic strengths in correspondence with the prior work using the reinforcement learning rules. However, we also present alternative interpretations in the discussion and expand upon the parameters adjusted as well.

### 2.2. Reward-Modulated Plasticity

The model put forth by Wei and Webb (2018a) focuses on a dynamic model of synaptic strength modulated by reward. Crucially, here we generalize this model of plasticity to be applicable to any parameter of the neural circuit more generally:


(3) $$\begin{array}{c} p(t)=A\text{ sin}\left(2\pi \frac{t\;-\sum_{0}^{k}T_{k}}{T_{k}}\right)\;+\;C\;if\;T_{k}<t<T_{k+1} \end{array}$$

where *p*(*t*) represents the parameter of interest, either a weight (*w*_*ij*_) or a bias (θ_*i*_), *t* is the current time, *A* is the amplitude of the parameter's fluctuation, *T*_*k*_ is the *k*th period length, and *C* is the center (“true center”) of parameter's fluctuation. As in Wei and Webb (2018a), when a parameter's fluctuation crosses its center while increasing, a new period is calculated using a Gaussian variable:


(4) $$T_{i}\;\sim \;N(\mu ,\omega^{2})$$

where μ is the center of the random distribution and ω^2^ is the variance. The fluctuation center *C* is updated according to the reward and the current value of the fluctuating parameter:


(5) $$\begin{array}{r} Ċ=\alpha \left(\;p\left(t\right)\;-\;C\right)\;R\left(t\right) \end{array}$$

where α is the learning rate and *R*(*t*) is the reward at time *t*. This results in the “true center” of a parameter being moved in the direction of the current displacement (whether the fluctuation above/below center) when reward is positive and in the opposite direction when the reward is negative. If the reward is precisely zero, the fluctuation center does not shift. The fluctuation amplitude *A*, is updated according to:


(6) $$\begin{array}{r} Ȧ=-\beta \;R\left(t\right) \end{array}$$

where β is the convergence rate. This enables the explore/exploit strategy: when the reward is positive, the amplitude of the fluctuation shrinks; when the reward is negative, the amplitude of fluctuation grows to explore a wider space. If the reward is precisely zero, the amplitude does not change.

### 2.3. Rhythmic Pattern Generation Task

Rhythmic patterns are one of the most commonly studied neural activities in the neuroscience literature, involved in a wide range of behaviors, from breathing to walking. To evaluate the effectiveness of the reinforcement learning rule on the dynamical recurrent neural circuit, we trained the circuits to produce rhythmic patterns. The performance on the task was measured by calculating the average change in neural output over time. The specific calculation of a circuit performance is given by the equation:


(7) $$\begin{array}{c} P(t)=\sum_{j=1}^{N}\frac{\left|o_{j}(t)\;-\;o_{j}(t^{\prime })\right|\;}{N} \end{array}$$

where *P(t)* is the performance at time *t, N* is the number of neurons,*o*_*j*_(*t*) is the output of neuron *j* at time *t*; and $\;o_{j}\left(t^{\prime }\right)$ is the output of neuron *j* at the previous time step. We consider the output of a neuron as: *o*_*j*_ = σ(*y*_*j*_+θ_*j*_). The task is performed without any sensory input (i.e., central pattern generator). The only way a circuit can maximize this function is by increasing and decreasing its outputs repeatedly; moving in only one direction will result in saturation and therefore stagnation in the fitness. As a result, optimizing the function results in oscillatory behavior. This task does not require oscillating at any specific frequency. However, given the way we define performance, larger amplitudes and faster frequencies result in increased performance.

### 2.4. Training Strategies

It is hard to gauge the effectiveness of a lifetime learning model when it is the only lifetime learning model that operates on a dynamical recurrent neural network. In order to examine of how well this lifetime learning mechanism searches the parameter space of the system, we compare it against two traditional search methods: a random walk search and a hill-climbing search. Although these two methods are not lifetime learning models (i.e., they are not a form of continuous, online training), they do provide a means of sampling the nearby parameter space of a given configuration. For the hill-climbing strategy, the relative increase in performance can be used to guide the search process. Our expectation for a lifetime learning mechanism is that it should be more efficient than a random walk, and at least as efficient as a hill-climbing search. This is based on the idea that as the system explores regions of parameter space that produce better performance, the lifetime learning mechanisms should guide the system toward those regions.

#### 2.4.1. Random Walker

As a baseline comparison, we use a random walk to search through the parameter space of synaptic weights from a random starting location. In every iteration, a small random number chosen from a uniform distribution is used to adjust each of the current weights of the system. The fitness of the new modified circuit is evaluated and recorded, and then a new random step is taken and the whole process is repeated. Although a random walker does not have a memory of where it found the best location, we report on the highest fitness value obtained throughout the random walk. The only parameter that affects the search in a random walk is the size of the step.

#### 2.4.2. Hill Climber

A hill climbing search operates similarly to a random walk. From a starting configuration, a random step is taken in parameter space. Unlike for the random walker, in a hill climbing search, the step is only consequential if the new configuration of the circuit performs better than or equal to the previous location of the circuit in parameter space. If the new point in parameter space performs worse than the original point, then a step in a new random direction is generated, and this process is repeated. As with the random walker, the only parameter that affects the search is also the size of the step.

### 2.5. Fitness and Reward Functions

In traditional approaches to training dynamic recurrent neural circuits, such as evolutionary algorithms, a single final performance on the task is provided as a fitness metric. Since such learning strategies do not explore the parameter space *during* evaluation, this is the only guidance they require. In contrast, for a reinforcement learning mechanism, the neural circuit must receive a reward signal based on changes in performance over time. After significant exploration of possible reward functions, we found that taking the difference between the instantaneous performance and a running average performance was effective. The reward function is defined as:


(8) $$\begin{array}{r} R\left(t\right)=P_{r}\left(t\right)-P\left(t\right) \end{array}$$

where *R*(*t*) is reward at time *t*, *P*_*r*_(*t*) is the running average at *t*, *P*(*t*) is the performance at *t*. The running average performance is calculated by taking the average value of the performance in some recent number of steps, which we called the sliding window size:


(9) $$\begin{array}{r} P_{r}\left(t+1\right)=\overset{Z}{\underset{j=1}{\sum }}\frac{P\left(t-j\right)}{Z} \end{array}$$

where *Z* is the sliding window size, which determines how much of the recent history is included in the running average calculation.

### 2.6. Evolving and Perturbing Solutions

In order to test the learning ability of the RL-CTRNN to perform the task, we wanted to ensure the task was solvable. The ability of the network to solve the task is contingent on the biases in addition to its synaptic weights. Thus, in order to be sure the task was solvable *via* changes in synaptic weights alone, we first evolved a high-performing solution using a Microbial Genetic Algorithm (Harvey, 2011). We then perturbed the weights of that high-performing solution in order to create different challenges depending on how much and how many weights were perturbed. For simplicity, we also fixed all time constants to be 1.0, since they do not change the actual dynamics of the network, just the timing scale. For consistency, when comparing different groups, we always used the same sets of starting configurations.

## 3. Results

In this section, we report on the results of a series of experiments to investigate the learning characteristics of the RL-CTRNN. We begin by looking in detail at a specific learning trajectory of a given RL-CTRNN. We then repeat the learning process multiple times from a common starting location and compare to repeated learning trials using alternative training strategies. After this we explore the ability of the RL-CTRNN to learn from a variety of locations in parameter space. Next we offer insight into the roles that different metaparameters play and how they break down. Finally, we report preliminary results on the scalability and generalization of the learning rules for training dynamical systems.

### 3.1. Result 1: RL-CTRNN Can Solve the Task

**Figure 1:**
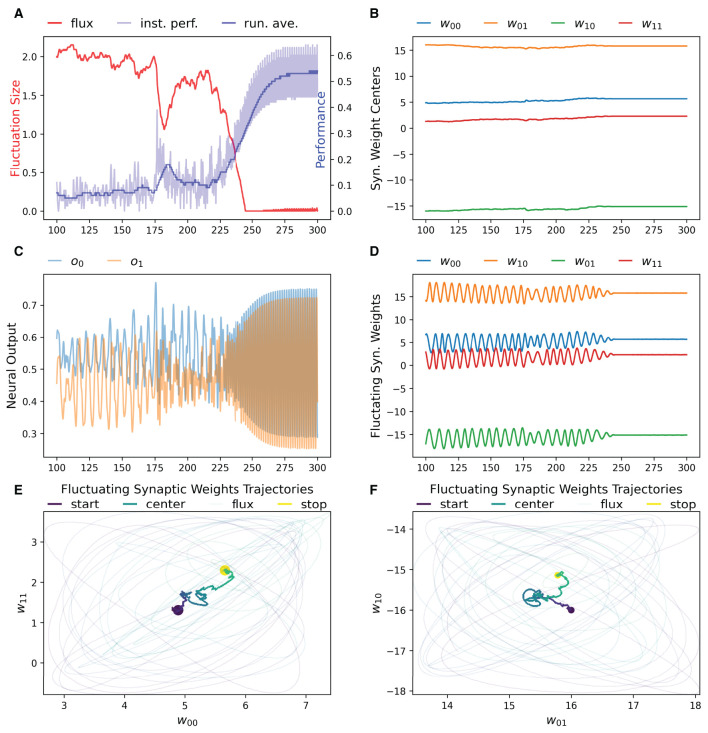
RL-CTRNN learning oscillatory behavior. Starting with a CTRNN configuration that does not oscillate, RL rules adjust the parameters (after allowing 100 s of transient dynamics to pass) until the CTRNN produces a high-performing, oscillatory behavior. **(A)** The instantaneous performance (light) and running average performance (dark) are shown in blue. The difference between these two determines the reward. The weight fluctuation (red) eventually decreases and converges. **(B)** Synaptic weights centers do not have to shift much, but eventually settle at a high-performing configuration. **(C)** Neural outputs over time. Initially there are some changes in output, but not smooth or fast. After 250 s the neural outputs are rapidly and smoothly oscillating. **(D)** Fluctuating synaptic weights over time. Initially the weights are fluctuating ± 2 (red line in **A**), but as performance improves the amplitude shrinks, although it briefly increases again around time 175, when the performance degrades briefly before improving again. By time 250, the weights have converged to a steady state. **(E,F)** Fluctuating synaptic weights trajectories. Circles indicate starting and stopping locations, the synaptic weights centers are shown relative to one another with a thick line and the fluctuating weights with thin lines. The fluctuations sample parameter space in the immediate vicinity of the centers and can guide the centers toward regions of higher performance.

**Figure 2:**
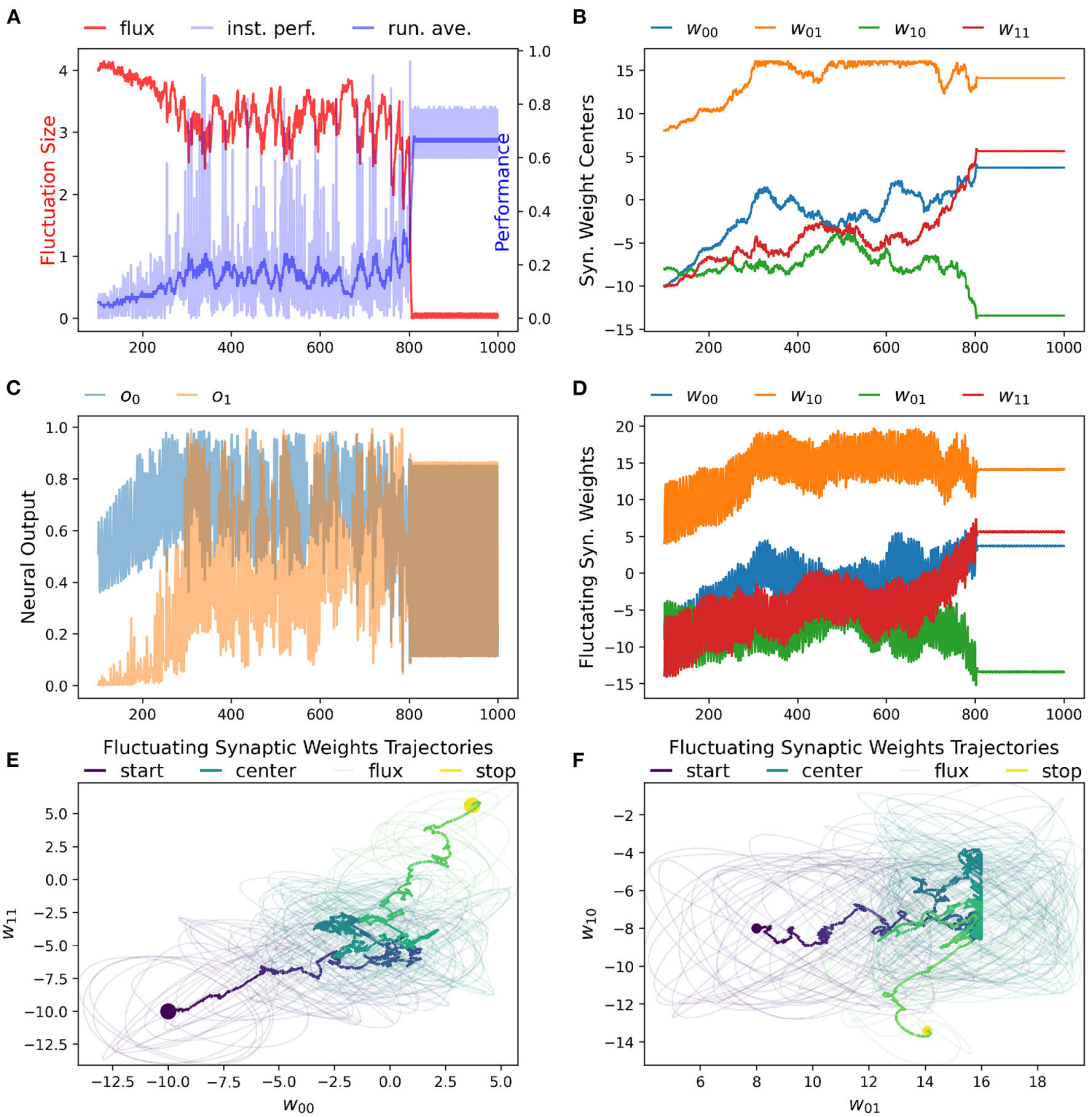
RL-CTRNN learning to perform oscillatory behavior from a distance starting configuration. **(A)** The size of synaptic weight fluctuation repeatedly shrinks as the performance improves and expands as the performance declines. **(B)** The synaptic weights centers have to move considerably to find a good combination that performs well. **(C)** Neural outputs over time. One neuron's output is initially fixed and inactive. Gradually both neurons become increasingly active. By time 900 the neural outputs are rapidly and smoothly oscillating. **(D)** Fluctuation of synaptic weights over time. Initially the weights are fluctuating ±4, but as performance improves the amplitude shrinks. By time 900, the weights have converged to a steady state. **(E,F)** Fluctuating synaptic weights trajectories over time. The weight centers appear to have nearly-linear initial and final trajectories—seemingly guided by the learning rule. The middle portion of the trajectory appears to be less directed, but eventually the network converges on a high-performing solution.

We have found that the RL-CTRNN is capable of solving the task when it is both close to and far from potential solutions. Figures 1, 2 show how the underlying CTRNN parameters change as well key controlling variables in the RL rules. Figure 1 shows the RL-CTRNN learning to oscillate when close to possible solutions. Figure 2 shows the RL-CTRNN learning to oscillate when significantly far away from possible solutions. In both cases, after an initial period of 100 s to allow transient dynamics to dissipate, the network switches back and forth between exploration and exploitation until arriving at a high-performing solution.

### 3.2. Result 2: Comparing the RL-CTRNN to Baseline Approaches

**Figure 3:**
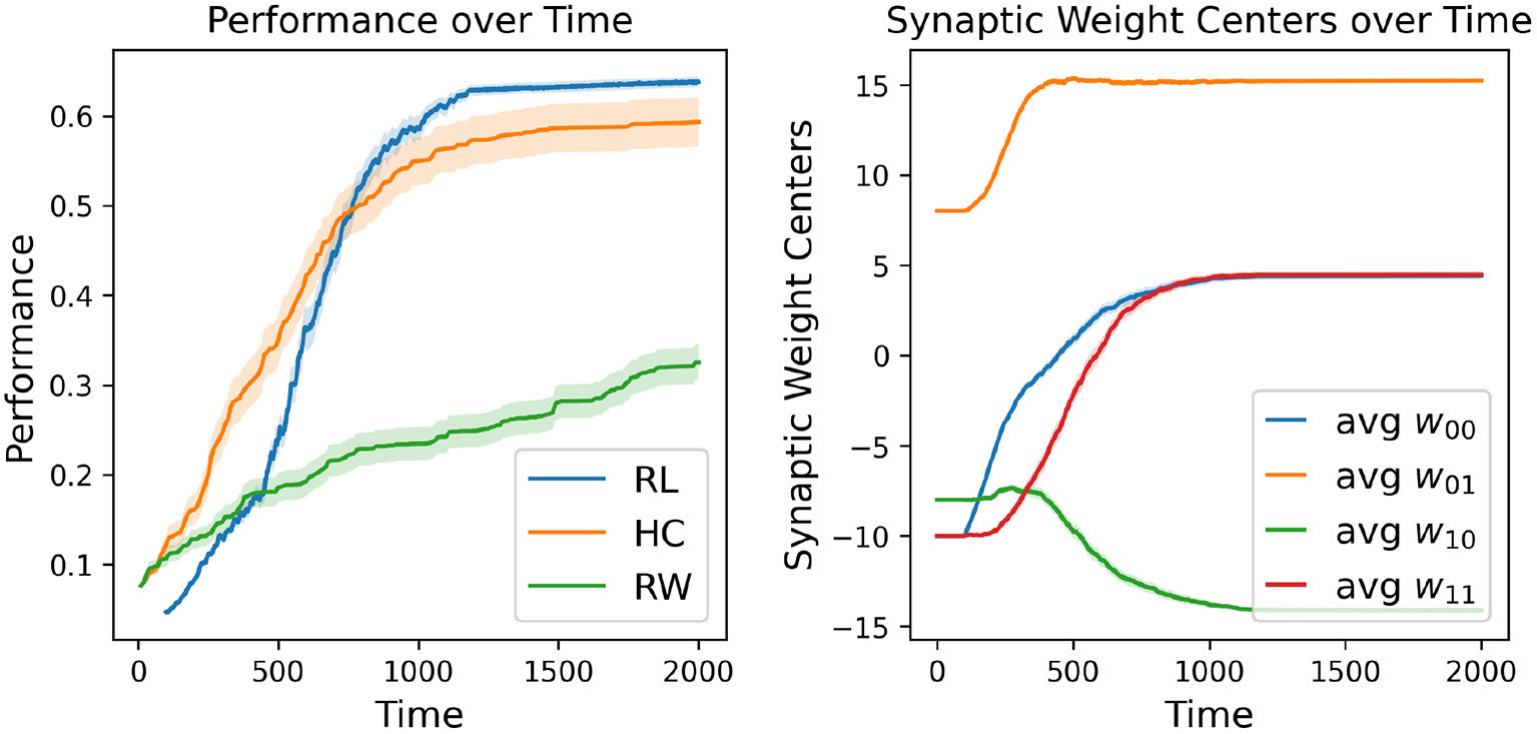
Learning strategy comparison. One hundred different runs were conducted from a single starting configuration for all individual learning strategies including random walking (RW), hill-climbing (HC), and the RL-CTRNN model (RL). The RW performance reported is simply the highest fitness score encountered during its entire trajectory (similar to the HC). The right plot shows the mean (with standard error—too small to see) synaptic weight centers of the 100 RL trained networks, which appear to have a highly robust trajectory despite being stochastic in nature.

The first major result of our experimentation was the demonstration that our RL-CTRNN model can successfully solve the task under certain constraints. However, with the knowledge that it is a largely stochastic process, we wondered how consistently it could do so. To investigate we decided to repeatedly test if a RL-CTRNN could learn from the same starting location. In addition, to assess characteristics of learning efficiency and robustness, we used our baseline models for comparison. Figure 3 shows how the individual learning strategies compare to each other over simulated time.

A direct comparison of which is superior here is not appropriate because there are a large number of constraints, metaparameters, and fundamental differences between these learning strategies. Nevertheless, we tried to make a meaningful comparison by making the relative rates of exploration similar: the mutation size of the hill-climber (HC) and random-walker (RW) is ± 8 and the initial fluctuation size is 4 (amplitude 8) for the RL-CTRNN. For reference, when the mutation size for the HC is set to ± 4, the HC learning process is greatly slowed. When the experiments in Figure 3 continued beyond time 2,000, eventually all the strategies would arrive at high-performing solutions. This particular view makes it easier to see the relative slopes of the different strategies when they are most clearly separated.

In addition, there are other metaparameters which could be set to increase the learning efficiency. However, in particular for the HC and RW these are less meaningful as a comparison to the RL since they involve very large jumps in the parameter space (i.e., the analogy of a trajectory through parameter space is lost when mutation sizes are very large, which tends to help these strategies). In the results presented, the RW and HC strategies utilize a 10 simulated second fitness function to determine the performance. The performance for the RW and HC are depicted on a scale of time based on how long it takes to evaluate an individual CTRNN configuration. In contrast, the RL-CTRNN learns online with a continuous reward signal based on its ongoing performance, thus it keeps updating throughout the entire duration of its simulation.

**Figure 4:**
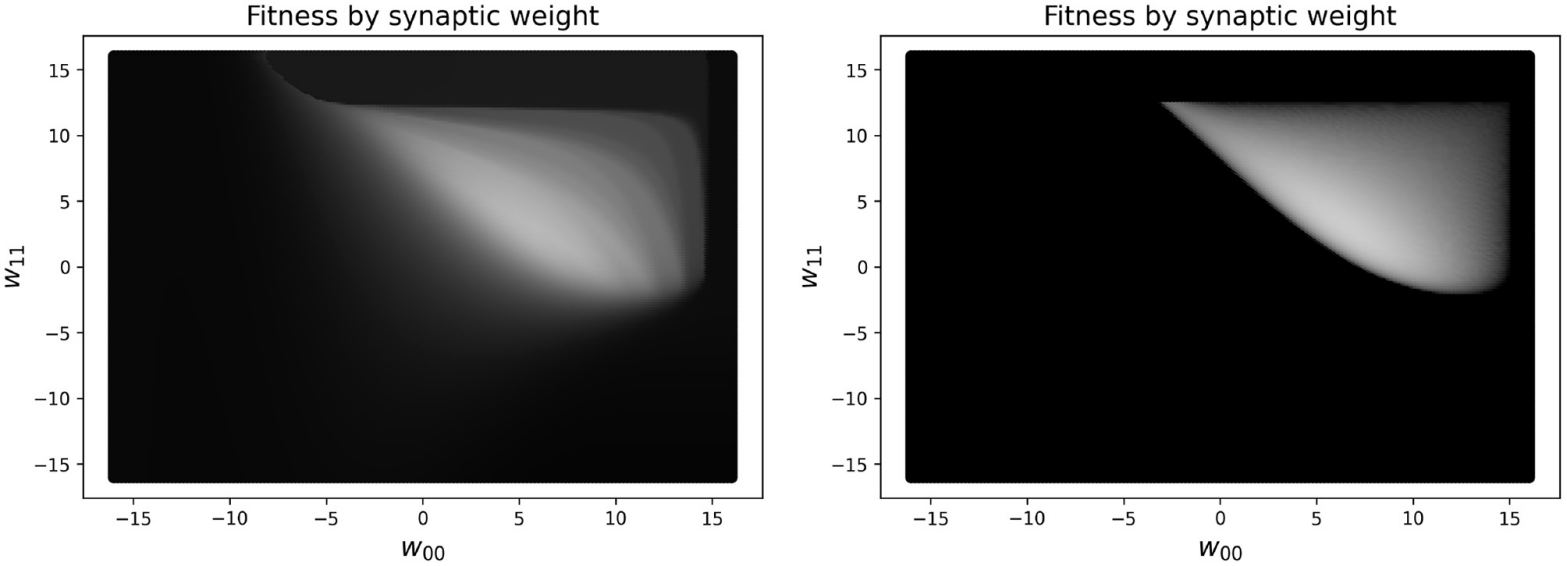
Landscape of a high-performing two node CTRNN while varying two specific weights (self-connections), which are closely related to one another. **Left**: Fitness in parameter space varying two self-connection weights using a 10 s performance metric, which includes initial transient dynamics as part of the measure of performance. **Right**: Fitness in parameter space varying two self-connection weights using a metric which only includes changes in outputs after 250 s have passed, in order to ignore the initial transient dynamics. The brighter the color, the higher the fitness. As can be seen clearly in the image, the left plot has a much larger region of the parameter space with intermediate shades of gray that would enable a learner to navigate from the darker regions to the lighter regions. In contrast, on the right there are large regions of black space which do not have a discernible gradient because there are minimal to zero ongoing oscillatory dynamics measured when calculating fitness.

In practice, reducing the simulated time to measure the performance for the RW and HC to less than 10 s makes the measure of the performance of the network unreliable. One concern is that this may result in a network not truly oscillating, but instead exploiting large initial transient dynamics. In fact, there are some (rare) cases where this might still occur, but having some transient dynamics present in the fitness measure is helpful for guiding the network toward higher performing solutions. To see a visualization of this, see Figure 4, which shows the difference in a slice of the fitness landscape (of a specific CTRNN configuration, which we reuse several times), which contrasts one fitness function including transient dynamics and one which excludes them.

Another significant difference from the RL-CTRNN is that the HC strategy is always allowed to start from a consistent state (initialized to zero), whereas the RL must deal with ongoing changes to the current state of the neurons in its system, including potentially challenging ones to recover from. The RL-CTRNN shows evidence of being robust at dealing with such challenges. In contrast, when the HC strategy requires the state to be maintained from the end of one mutation's simulation to the start of another (similar to the ongoing process in the RL-CTRNN), it struggles and in some cases performs worse than even the RW.

One of the advantages of the RL-CTRNN over the HC learning strategy is that the fluctuation size, i.e., how much the synaptic strengths vary over time, is controlled by the reward signal, meaning it can increase or decrease. This would be equivalent to a mutation size that can shift over time, much like other processes such as simulated annealing (Kirkpatrick et al., 1983), in which an initial exploration phase slowly is shifted toward exploitation.

### 3.3. Result 3: Analysis of RL-CTRNN Learning From a Variety of Starting Configurations

**Figure 5:**
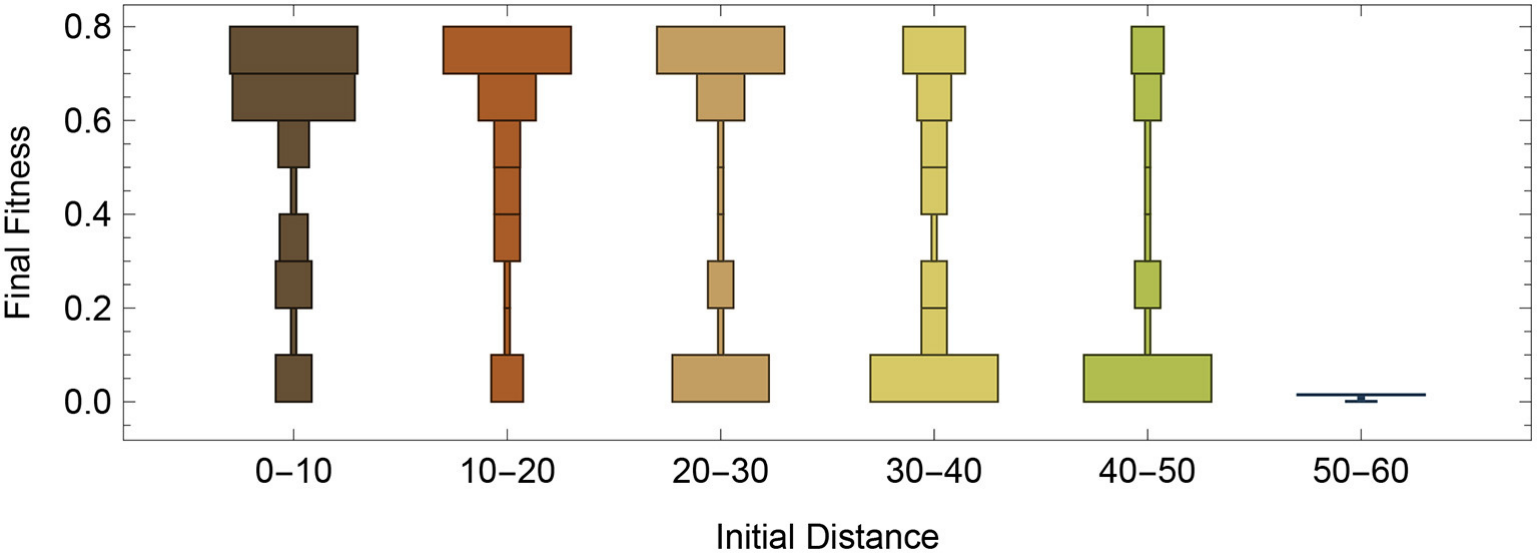
Performance as a function of initial distance to a good solution. Systematic testing of different regions of entire parameter space, including 9 samples from each dimension for 6,561 different starting configurations. Each RL-CTRNN was allowed to run for 10,000 simulated seconds.

Utilizing the same starting configuration from Figure 2, we decided to do a systematic evaluations of how well the RL-CTRNN could learn to oscillate from across the entire synaptic weight parameter space. To do this, we varied the 4 synaptic weights from −16 to +16 in intervals of 4. After analysis of a variety of factors, we determined that one of the most predictive factors in the success of the RL-CTRNNs was their initial distance from the original high-performing solution. Figure 5 shows that there tend to be a bi-modal split (Figure 5, 30–40 and 40–50) in performance, with the majority of networks close to the original solution attaining it and in the most extreme distance, none of the RL-CTRNNs being successful. It is likely that even in the farthest distances, that the RL-CTRNNs could eventually learn to oscillate, but that the 10,000 simulated seconds provided were insufficient. In fact, additional analysis of the data indicated even those with a low final fitness had moved in the direction of high-performing solutions. Interestingly, there are some starting configurations that began close to the original (Figure 5, 0–10) known solution, that ended up having a low fitness. Understanding why this happens requires a deeper investigation into some of the metaparameters of the model, which we will investigate and discuss next.

### 3.4. Result 4: Metaparameter Analysis

**Figure 6:**
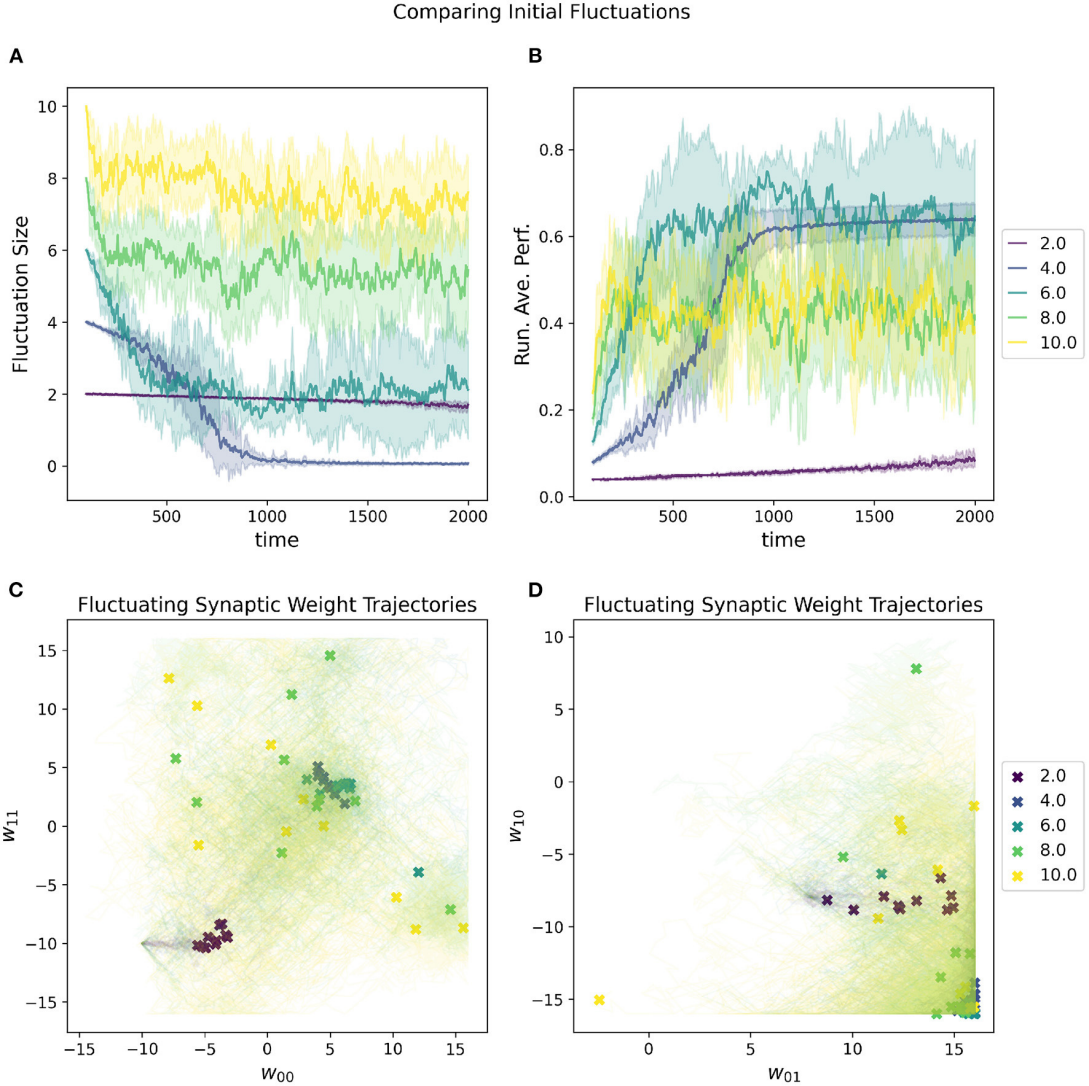
Impact of initial fluctuation size on a RL-CTRNN's learning trajectory. Ten runs were conducted for each value of the metaparameter and then aggregated results are shown. **(A)** The different initial fluctuation sizes show very different dynamics over time, some converging, some partially converging, and some barely converging at all. **(B)** The running average performance stabilizes when the fluctuation size converges toward zero. **(C,D)** The final locations of the various instances of the different values of the initial fluctuations are given by X marks and the trajectories of the weight centers are shown with light thin lines of the same color. The larger initial fluctuations (yellow, green) trajectories end (but don't converge) in a wide range of end locations, some including the high fitness, but not consistently. The locations of high-performance are consistent with the white regions (high fitness) of Figure 4 and are where successful solutions ended.

A random selected starting location in parameter space most will almost certainly have a very low initial performance. One of the metaparameters of the RL-CTRNN is the initial fluctuation size for the synaptic weights. As the RL-CTRNN adapts and performance increases, the fluctuation's amplitude shrinks until the network converges and stops exploring the parameter space. This is an inherent challenge for any learning strategy, which is, “How do I know when to stop learning?” The setting one provides to the initial fluctuation is effectively an estimate of how much the network expects to increase its performance from beginning to end. The effects of varying this metaparameter can be seen in Figure 6. When its initial fluctuation size is too small, the RL-CTRNN does not shifts its weights far before its fluctuation amplitude shrinks, which limits how far it can travel. This is because the rate at which it changes depends on the distance a fluctuating synaptic weight is from its center. In contrast, when the initial fluctuation is too large, the network moves quickly, but does not converge quickly enough in a region of high fitness.

The advantage of a larger initial fluctuation size is the greater ability to explore the parameter space, however, as the fluctuation increases in amplitude, the rate at which the network is “moving” through parameter space increases as well. A consequence of an increased “speed” through parameter space is that it is possible to move through a “good" region of the parameter space so quickly that the network does not have a chance to slow down rapidly enough to remain in the “good” region. This “overshooting” appears to be the case for some of the large values depicted in Figure 6 as well as mentioned earlier (Figure 5, 0–10). In particular, despite appearing to enter regions of high-performance, the network does not converge.

**Figure 7:**
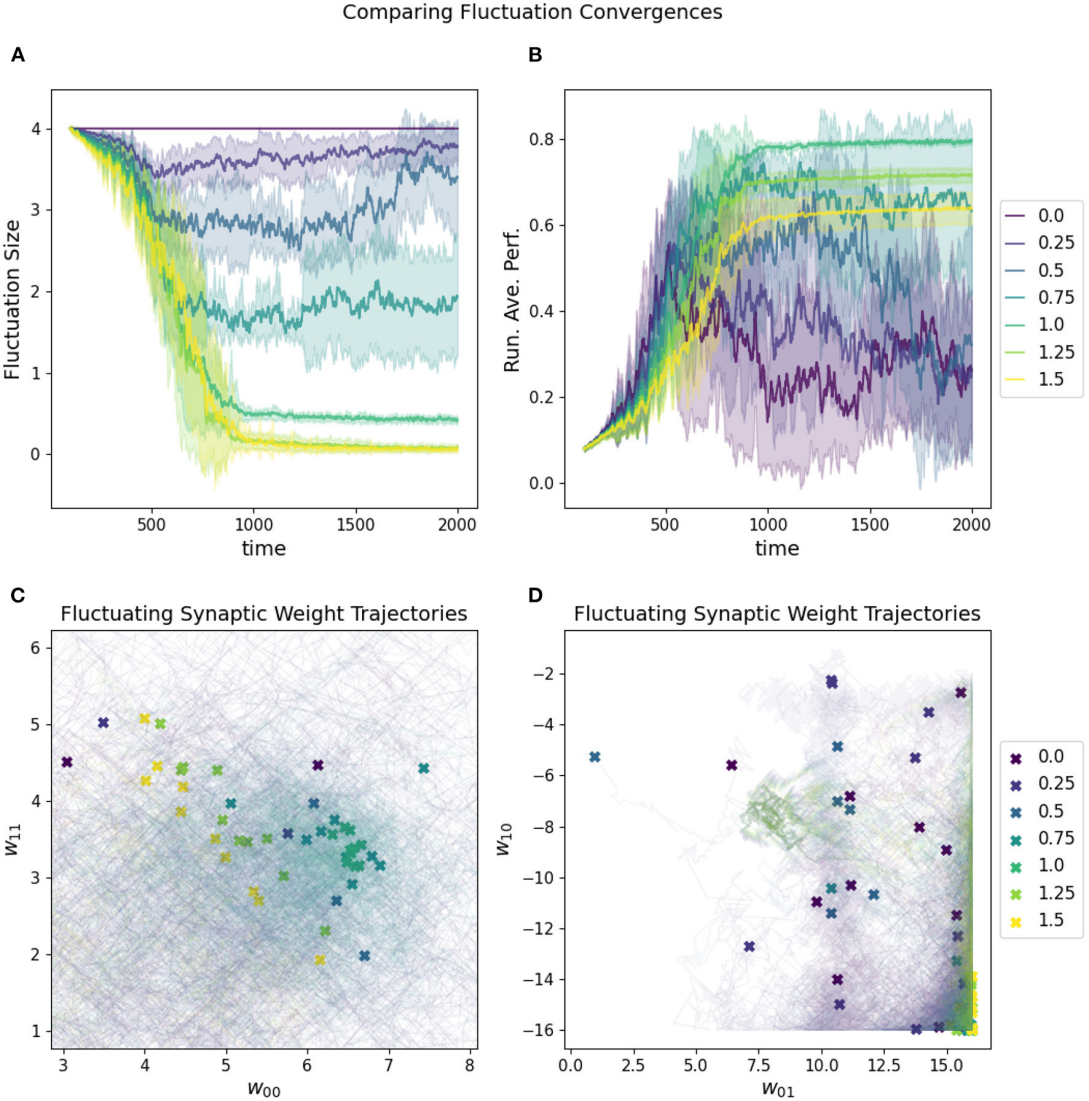
Impact of convergence rate on a RL-CTRNN's learning trajectory. Ten runs were conducted for each value of the metaparameter and then aggregated results are shown. **(A)** All start with an initial fluctuation size of 4, but those with larger convergence rates more rapidly converge and stop moving through parameter space. **(B)** The running average performance stabilizes when the fluctuation amplitude converges toward zero, note that the faster convergence does not necessarily result in the highest final performance. **(C,D)** The final locations of the various instances of the different values of the fluctuation convergence are given by X marks and the trajectories of the weight centers are shown with light thin lines of the same color. **(C)** is zoomed in on a particularly dense region of final configurations and it is possible to see how the faster converging solutions did not move as far into the direction of the higher performing runs. In other words, they prematurely converged.

The rate at which the network increases and decreases its fluctuation amplitude is the convergence rate, which must be aligned properly with the fluctuation amplitude if the “overshooting” problem is to be avoided (see Figure 7). One reasonable constraint is to set the initial fluctuation size to be the smallest value which produces a reasonable chance of finding solutions to a given target problem. Again, this is necessarily related to the challenge of the particular parameter space of a given task.

**Figure 8:**
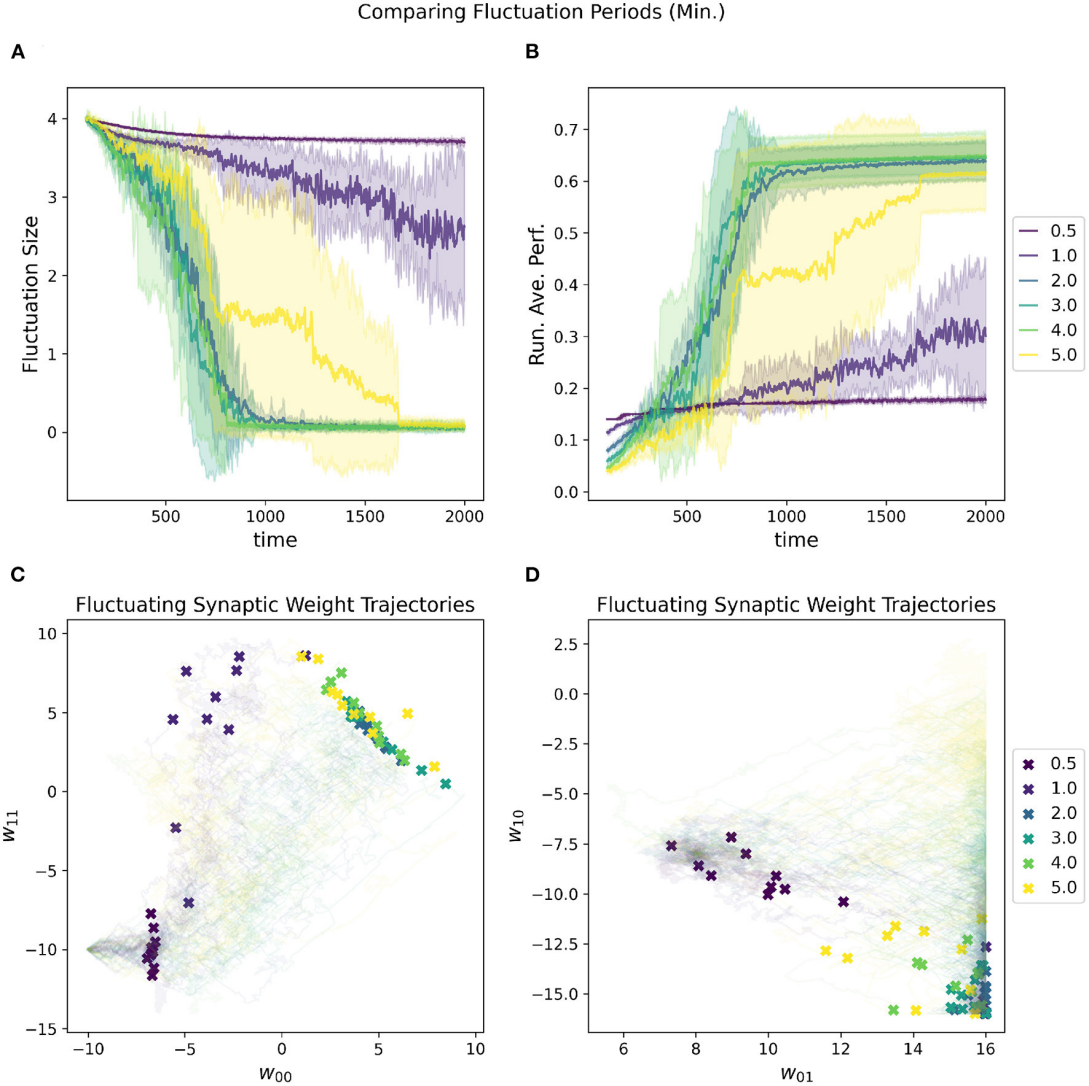
Impact of minimal fluctuation period length on a RL-CTRNN's learning trajectory. Ten runs were conducted for each value of the metaparameter and then aggregated results are shown. **(A)** All start with an initial fluctuation size of 4, the RL-CTRNN struggles to move in the correct direction when the periods are too short. **(B)** The running average performance stabilizes when the fluctuation amplitude converges toward zero, note that the faster convergence does not result in the highest final performance. Longer periods present more stable trajectories, but as they grow longer, they reduce the speed at which the network can sample and hence move through the parameter space. **(C,D)** Results indicate that for one group of learners with a minimum fluctuation period some of the weights approached the known high-performance region **(C)**, but other synaptic weights were did not reach the high-performance region (lower right of **D**).

In addition to the initial fluctuation amplitude and the convergence rate, the period of the oscillations also contributes to the speed at which the network is moving through parameter space. If the periods are shorter, then the network moves faster with the increased risk for overshooting “good” regions of parameter space, especially as fluctuation amplitude is high. Figure 8 depicts the impact of adjusting the estimated minimal period length [estimated based on the variance subtracted from the mean of the distribution (Equation 4)]. The estimated maximal period length is set to be double the minimum.

However, there is an inherent trade-off between robust performance and efficiency with the length of periods. In general, the shorter the period, the more of the parameter space can be explored since there are more trajectories through the space. With the increased speed and efficiency comes the risk of “overshooting.” Thus, one must be sure that the period is long enough to allow convergence to a solution, but not so long that there are not enough trajectories to find a solution. There is another critical consideration with respect to the periods, which is that the RL rule depends upon the oscillations of the parameters being slow enough that when the reward is updated that the network is still in close proximity to the region of parameter space that resulted in the highly rewarded behavior (this is consistent with Wei and Webb, 2018a). For our task, the reward signal is continuous and thus gives a chance to have a relatively fast period since the reward can be quickly updated. However, the reward signal is constantly fluctuating up and down due to the nature of the oscillating behavior of the circuit (just as the rate of change slows at the top and bottom of a sine wave), making it difficult for the network to converge on a solution.

To help address this concern, we found that having a running average for the performance of the circuit was helpful. In particular it was useful to compare the instantaneous performance to the running performance in order to calculate a reward signal. This is somewhat analogous to the reward prediction error hypothesized to be provided by dopamine and commonly used in reinforcement learning (Glimcher, 2011). In other words, we chose to provide a reward signal to the agent based upon how much better it is currently doing than it has recently done. This helps to guide the network toward increasingly better regions of parameter space or toward exploration away from worse regions.

**Figure 9:**
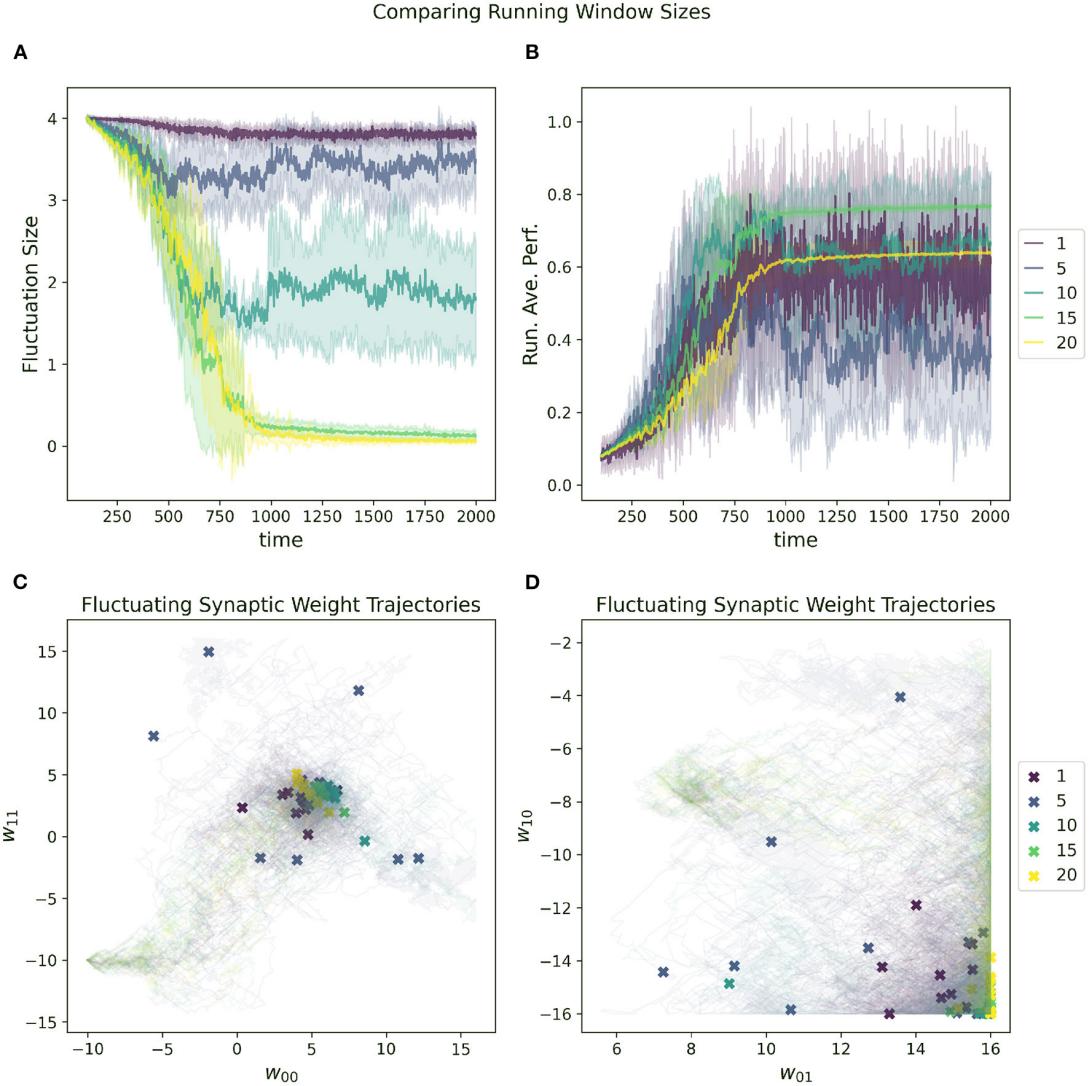
Impact of running average performance window size (*Z* in Equation 7) on a RL-CTRNN's learning trajectory. **(A)** All start with an initial fluctuation size of 4, the RL-CTRNN struggles to converge when the sliding window size is too short. **(B)** There appears to be a minimum window size sufficient to allow successful convergence. **(C,D)** For the small values of the sliding window size, the trajectories appear to move sufficiently far through parameter space, but have trouble converging in high-performing regions.

The impact of increasing or decreasing the size of this sliding window can be seen in Figure 9. In general, the RL-CTRNN can learn successfully with a wide range of window sizes as long as they are sufficiently large. With an increased window size, the rate at which the running average changes slow down and since the reward is based on the difference between the instantaneous performance and the running average performance, the potential for premature convergence increases as well. This can be seen in 9A and 9B, where the convergence happens at the same time for window size 15 and 20, but the final performance with window size 20 plateaus early.

### 3.5. Result 5: Scalability and Generalization

**Figure 10:**
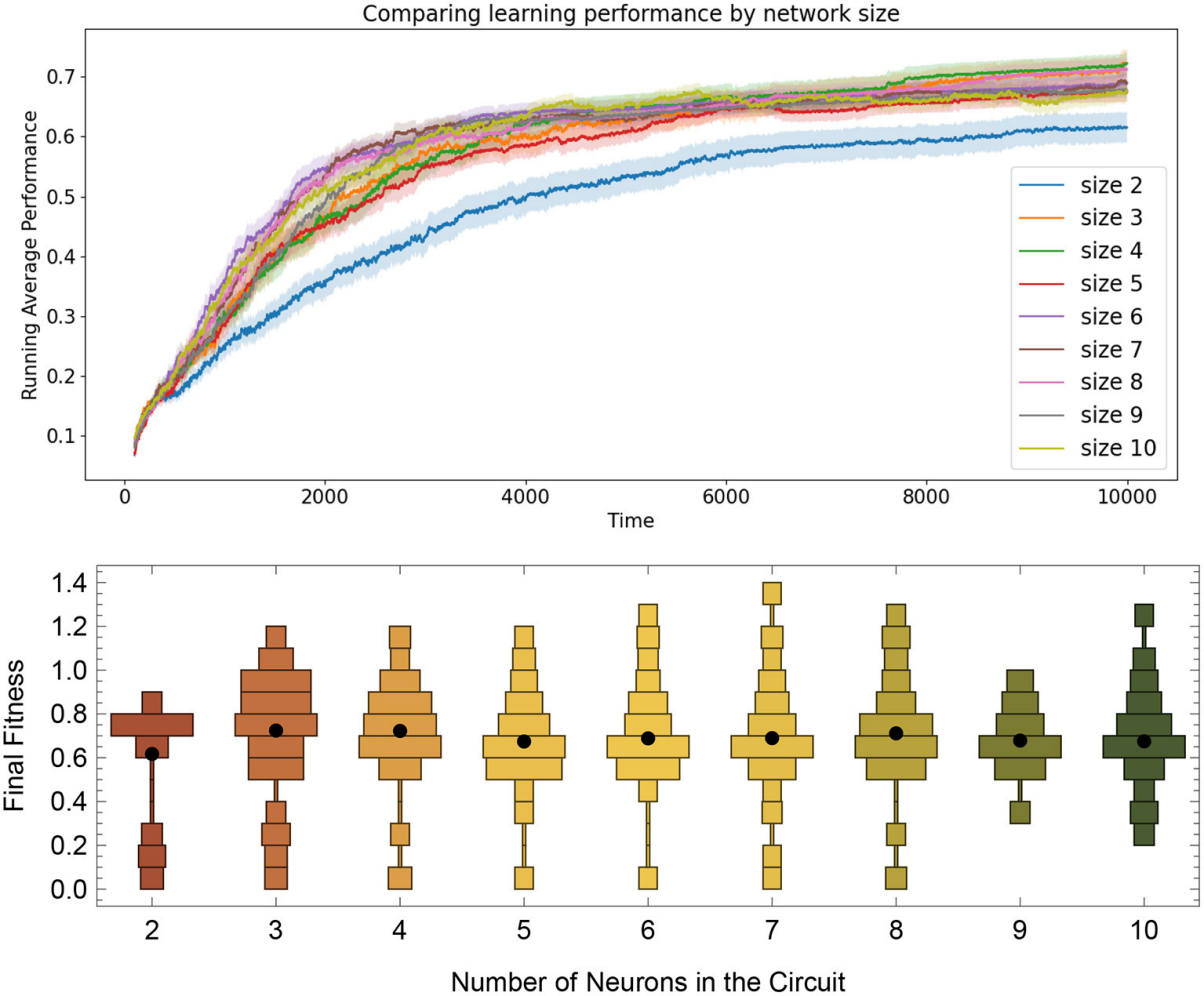
Comparing task learning from random starting configurations by network size. **Top**: The solid lines are mean performances of networks started from 100 random starting locations. Results show that the size 2 networks tend to have a smaller average fitness than the others, but that the rest of the network sizes up to size 10 all seem to arrive a solution at roughly the same rate, despite have the same metaparameters across all sizes. **Bottom**: Distribution of running average performance values at the end of the 10,000 simulated seconds for the 100 networks trained from size 2 to 10.

**Figure 11:**
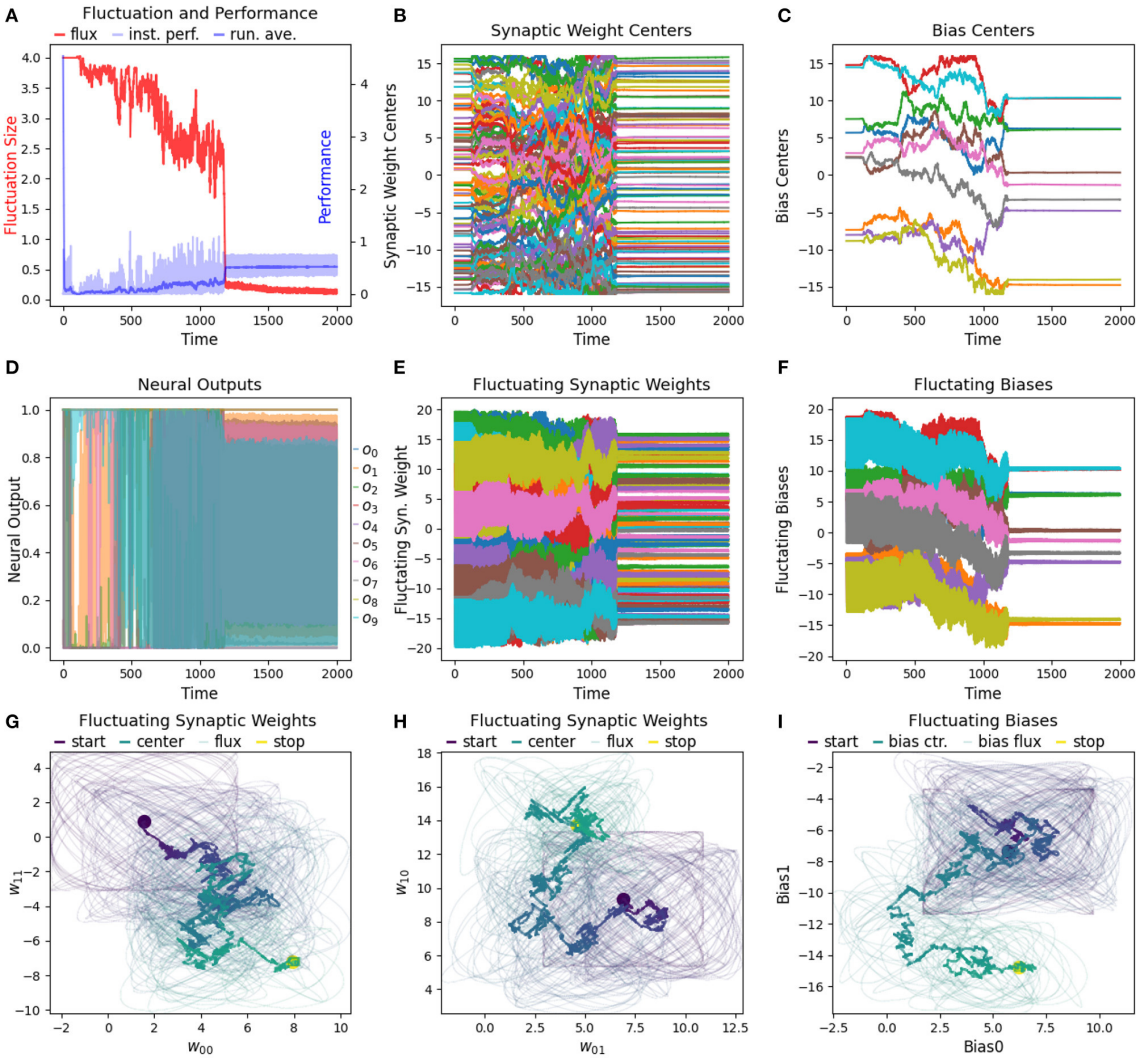
Detailed view of a single run of a size 10 network being trained from a random starting location. **(A)** After 1,000 s a RL-CTRNN with 10 neurons learns to oscillate and converges on a high-performing solution. **(B,C)** A total of 110 parameters are all simultaneously adjusted from the single continuous global reward signal. **(D)** Some of the neurons are not changing state, so we know that better solutions exist. However, we did not attempt to optimize any of the metaparameters for the different size networks. **(E–I)** The same rules for updating the synaptic weights *via* a single global reward signal are applied to the biases as well. Surprisingly, the biases here use the same fluctuation size and fluctuation period metaparameters without requiring any special adjustments.

To determine how well the RL-CTRNN learning scales, we determined that we could test two things. First, we wanted to expand the RL-CTRNN model to adjust the biases in a similar fashion to the synaptic weights. Second, we wanted to see if the network could be trained from a random starting location, given that the biases were trainable. Figure 10 shows the results of this experiment. To gain a sense of the challenge of using a single global reward signal to train a larger number of parameters simultaneously, consider the results in Figure 11, which shows that all 100 synaptic weights and 10 biases are simultaneously trained using the same single global reward signal.

## 4. Discussion

In this work, we have presented the application of the previously published reinforcement learning rules to a neural model (CTRNNs) commonly used in computational neuroscience. Previously, CTRNNs have been trained predominantly *via* evolutionary algorithms. This work demonstrates that the RL rules can successfully be applied to train CTRNNs. We also provided a baseline comparison to two simple learning strategies and reported on general characteristics of the efficiency and robustness of the RL rules for CTRNNs. Our results demonstrate that the RL-CTRNN can learn to perform an oscillatory behavior from a variety of different initial configurations when given the proper set of values for metaparameters. We also provided insights into the ways that extreme values for certain metaparameters disrupt the learning process. In addition to our application of proposed learning rules to the CTRNN model, we have expanded and demonstrated the ability to train additional parameters beyond synaptic weights (we also trained biases) in dynamical models. Given the ease with which this was done, we expect it can easily be applied to additional parameters of dynamical systems.

Despite the demonstrated success, there are limitations to this initial study, the biggest perhaps being that we only used a single task, one which is ideally suited for rapid learning given a continuous high-fidelity performance metric (change in neural outputs, Equation 7). Our comparison to the HC learning strategy is limited in that it has a number of dissimilarities. However, we can see that the RL is effective at sampling a large number of regions in the parameter space *via* synaptic weight or bias fluctuations as opposed to the much longer and perhaps higher quality samples utilized by the HC.

This work opens up new possibilities for training CTRNNs specifically, but dynamical systems more generally. As mentioned in the Section 2, we originally considered the connections between nodes in the CTRNN as analogous to synaptic weights, however, CTRNNs can be used to approximate dynamical systems more generally, so there is no reason to limit this rule to just synaptic weights. We should expect many biological processes to include periodic variations at a variety of timescales. Given that the RL rules seems to operate well at a variety of timescales, it seems promising as a potential explanatory mechanism for additional learning processes in the brain or body yet to be discovered.

### 4.1. Future Work

We see several fruitful directions for future inquiry relating to additional tasks, different types of reward rules, synthesis with developmental brain/body models, and combination with evolutionary algorithms.

#### 4.1.1. Tasks

First, we see exciting opportunities to apply the RL-CTRNN model to a variety of the embodied, embedded tasks that CTRNNs have frequently been evolved for in the past. This is also exciting as it offers a chance to test out robustness and adaptivity *via* lifetime learning through oblation studies or additional dynamical tasks. There is also an opportunity to explore multi-functionality wherein a single controller is tasked with performing multiple separate tasks. To date, CTRNNs have been used to investigate multi-functionality through neural re-use (Candadai and Izquierdo, 2018), but not where the parameters can change as the tasks are being performed (outside of the neural dynamics themselves).

#### 4.1.2. Reward Rules

We were impressed with the ability to use a single global rule to train so many parameters simultaneously. This offers support to the idea that separate reward signals could also be used to train different parameters or components of a dynamical system. Although not explored yet, there might be advantages to adjusting the synaptic weights of a CTRNN at a different period, amplitude, or convergence rate than the biases of the same network. Furthermore, it might be possible to have multiple reward signals for different tasks such that the network could be trained to perform multiple behaviors simultaneously with mixed reward signals controlling the process, potentially at different timescales. A simple direction to start could be to train a large RL-CTRNN to maximize change in neural output as we did in this work, but to allow each individual neuron's change in output to serve as a reward for the incoming synapses to that neuron. This might make it possible to rapidly train all the neurons in the network to oscillate.

#### 4.1.3. Developmental Model

Given the seeming universality of the RL rules to train various components of dynamical systems, this learning rule appears to have potential to train systems which undergo significant structural change, such as through the addition or removal of components, whether in a body or a brain. Given this possibility, we foresee the potential for a developmental process in which a CTRNN, and potentially body, are allowed to slowly grow and then converge to an effective configuration using the fluctuating learning mechanisms explored in this article.

#### 4.1.4. Combining Lifetime and Evolutionary Learning

Increasingly, work combining evolution and lifetime learning mechanisms have been proposed and explored (Todd et al., 2020; Gupta et al., 2021) and this offers yet another opportunity to combine lifetime learning and evolution. The synergy of these two forms of learning might enable training dynamical systems previously too difficult to train using purely lifetime learning or evolutionary approaches alone. This could open more possibilities for modeling work in computational neuroscience.

## 5. Conclusion

Training dynamical, continuous neural networks is often challenging and traditional optimization methods such as back-propagation cannot be applied to these types of networks. In this article, we applied reinforcement learning-like rules to attempt to train Continuous-Time Recurrent Neural Networks to perform oscillatory behaviors. Results show that with the properly tuned metaparameters, the learning rules are efficient, robust, and can easily scale up to training a large number of parameters. These results suggest a number of exciting areas where this approach could be applied, which we presented in the discussion.

## Data Availability Statement

The raw data supporting the conclusions of this article will be made available by the authors, without undue reservation.

## Author Contributions

JY was the primary developer of the code, experiments, analysis, and writing. CA contributed to code, experimentation, and some analysis. CW contributed to experimentation and some analysis. EI contributed ideas, aided in producing figures, and provided revisions to the writing. All authors contributed to the article and approved the submitted version.

## Funding

This work was supported by NSF Grant 1845322.

## Conflict of Interest

The authors declare that the research was conducted in the absence of any commercial or financial relationships that could be construed as a potential conflict of interest.

## Publisher's Note

All claims expressed in this article are solely those of the authors and do not necessarily represent those of their affiliated organizations, or those of the publisher, the editors and the reviewers. Any product that may be evaluated in this article, or claim that may be made by its manufacturer, is not guaranteed or endorsed by the publisher.
